# Supplementary material for: A UMLS-based spell checker for natural language processing in vaccine safety
Source: BMC Med Inform Decis Mak. 2007 Feb 12;7:3. doi: 10.1186/1472-6947-7-3 (PMC1805499; doi:10.1186/1472-6947-7-3)
Supplement: Additional file 13 — RAP application directory. Sets up directory for RAP (RDF application for PHP) source code [file 1472-6947-7-3-S13.gz › rap/api/util/adodb/tests/test2.php]

Untitled

php
/\*
V4.52 10 Aug 2004 (c) 2000-2004 John Lim (jlim@natsoft.com.my). All rights reserved.
Released under both BSD license and Lesser GPL library license.
Whenever there is any discrepancy between the two licenses,
the BSD license will take precedence.
Set tabs to 8.
\*/
#
# test connecting to 2 MySQL databases simultaneously and ensure that each connection
# is independant.
#
include("../tohtml.inc.php");
include("../adodb.inc.php");
ADOLoadCode('mysql');
$c1 = ADONewConnection('oci8');
if (!$c1-PConnect('','scott','tiger'))
die("Cannot connect to server");
$c1->debug=1;
$rs = $c1->Execute('select rownum, p1.firstname,p2.lastname,p2.firstname,p1.lastname from adoxyz p1, adoxyz p2');
print "Records=".$rs->RecordCount()."  

```
";
//$rs->_array = false;
//$rs->connection = false;
//print_r($rs);
rs2html($rs);
?>
```
